# Mutational Signatures as Sensors of Environmental Exposures: Analysis of Smoking-Induced Lung Tissue Remodeling

**DOI:** 10.3390/biom12101384

**Published:** 2022-09-27

**Authors:** Yoo-Ah Kim, Ermin Hodzic, Bayarbaatar Amgalan, Ariella Saslafsky, Damian Wojtowicz, Teresa M. Przytycka

**Affiliations:** National Center for Biotechnology Information, National Library of Medicine, National Institutes of Health, Bethesda, MD 20894, USA

**Keywords:** mutational signatures, smoking, lung cancers, APOBEC, immune response to smoking, cell-type composition, goblet cells, ciliated cells, basal cells

## Abstract

Smoking is a widely recognized risk factor in the emergence of cancers and other lung diseases. Studies of non-cancer lung diseases typically investigate the role that smoking has in chronic changes in lungs that might predispose patients to the diseases, whereas most cancer studies focus on the mutagenic properties of smoking. Large-scale cancer analysis efforts have collected expression data from both tumor and control lung tissues, and studies have used control samples to estimate the impact of smoking on gene expression. However, such analyses may be confounded by tumor-related micro-environments as well as patient-specific exposure to smoking. Thus, in this paper, we explore the utilization of mutational signatures to study environment-induced changes of gene expression in control lung tissues from lung adenocarcinoma samples. We show that a joint computational analysis of mutational signatures derived from sequenced tumor samples, and the gene expression obtained from control samples, can shed light on the combined impact that smoking and tumor-related micro-environments have on gene expression and cell-type composition in non-neoplastic (control) lung tissue. The results obtained through such analysis are both supported by experimental studies, including studies utilizing single-cell technology, and also suggest additional novel insights. We argue that the study provides a proof of principle of the utility of mutational signatures to be used as sensors of environmental exposures not only in the context of the mutational landscape of cancer, but also as a reference for changes in non-cancer lung tissues. It also provides an example of how a database collected with the purpose of understanding cancer can provide valuable information for studies not directly related to the disease.

## 1. Background

Over the last few decades, the scientific community has continued to collect large quantities of biomedical data, typically organized in specialized databases. One such effort, The Cancer Genome Atlas (TCGA), a landmark cancer genomics program, includes data on over 20,000 primary cancer and matched normal samples, spanning 33 cancer types. As research questions continue to evolve, such historical data, combined with new computational approaches, remain fundamental for generating and testing new hypotheses and suggesting new experimental analyses.

Many lung diseases, including cancer, are associated with environmental factors, such as smoking or air pollution. Prolonged exposure to these factors often leads to chronic changes in lung structure and function. However, interactions between such environmental exposures and molecular-level changes in lung function are not fully understood. The amounts of environmental exposures are difficult to measure, making it challenging to quantify their impacts. In some cases, individuals might even be unaware of being exposed to harmful elements. Even when a sustained exposure can be established, as it is the case in smoking, the level of the exposure is often under-reported [1]. Furthermore, cigarette smoke contains a mixture of chemicals [2], and many factors, such as cigarette type, strength, and smoking habits, also contribute to the net exposure to individual factors. To bypass this challenge, studies typically resort to using binary classification—ever smoker vs. never smoker (e.g., [3])—even though continuous measurements could be more informative. The impact of cigarette smoking might also be indirect. For example, it is known that cigarette smoking is one of sources of chronic inflammation [4], which might in turn lead to chronic obstructive pulmonary disease (COPD) or cancer. Cigarette smoking has been also linked to differences in response to immunotherapy [5,6,7,8,9,10,11]. Thus, in order to better understand the process of emergence of lung diseases, it is important to develop computational approaches, which, while leveraging existing data, can help to untangle the impact of various factors on molecular changes in lung tissue. The emerging concept of mutational signatures can offer an interesting opportunity to uncover hidden relations between cellular level changes and a certain class of external exposures.

Smoking, and many other environmental exposures, are known to be mutagenic. The effects of such mutagenic exposures have been studied extensively in the context of cancer [12,13] and recent studies leveraged the idea of mutational signatures—characteristic mutation patterns imprinted on DNA molecules by specific mutagens [14,15,16,17,18]. Mutational signatures are typically defined based on a partition of mutations into mutation categories. Most studies utilize mutation categories defined based on six types of single nucleotide substitutions (C > A, C > G, C > T, T > A, T > C, and T > G), considered in the context of the 5${}^{\prime }$ and 3${}^{\prime }$ flanking nucleotides, yielding 96 mutation categories (e.g., TCC > TAC, and CAG > CTG). Given such categories, mutational signatures are defined as multinomial distributions of mutation counts over these categories. Following the pioneering paper of Alexandrov et al. [17], several computational methods have been proposed to infer such signatures based on large cancer datasets. The Catalogue of Somatic Mutations in Cancer (COSMIC) contains a reference set of signatures defined using the 96 mutation categories mentioned above. COSMIC signatures have been broadly explored and many, but not all, have been linked to specific mutagenic processes. A decomposition of somatic mutations in a tumor genome into COSMIC signatures and mutation counts attributed to each signature (signature exposure) can provide patient-specific information about mutagenic factors contributing to the somatic mutations in the tumor (reviewed in [19,20,21,22]).

Mutational signatures can be easily inferred from bulk genome sequencing of tumor samples. Although the influence of environmental factors, such as smoking, is not restricted to tumors but also affects the whole organism, mutations in non-cancer cells are not common and are difficult to capture by bulk sequencing since such cells are not related by common ancestry from a tumor-initiating cell. Since mutagenic processes caused by environment-related mutagens are exogenous for both cancer and non-cancer samples, signature exposures inferred based on cancer mutation data can be used to estimate the strength of the corresponding environmental factors acting on non-cancer cells as well. However, while considering environmental processes through the lenses of mutational signatures provides unique opportunities, it also comes with its set of challenges. Some environmental factors, including cigarette smoke, are mixtures of many potentially harmful components. While some such components might be uniquely associated with smoking, others might be present in other contexts as well. In addition, even if the sample itself is non-neoplastic, it should not be ignored that the sample donor was a cancer patient. Conveniently, in many cancer types, tumor growth is correlated with a specific mutational signature (SBS1), allowing for pinpointing correlations that could be due the disease’s status rather than environment. Finally, the etiologies of many mutational signatures are not fully understood, and not all chemicals impacting cell function are mutagenic but might instead co-occur with mutagenic exposures. Thus, as in any association-based analysis, additional studies might be required to obtain mechanistic explanations of the uncovered associations (see Section 3).

**Figure 1:**
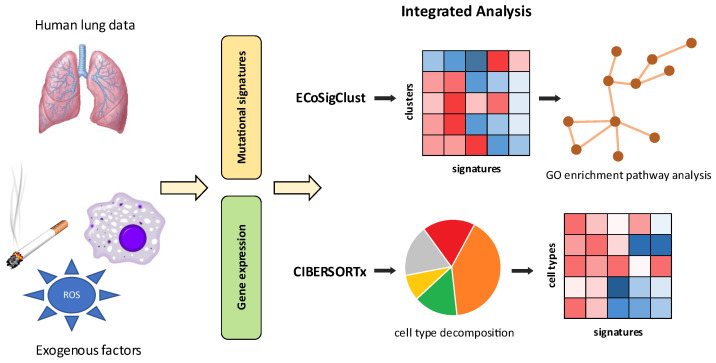
The analysis overview of the impact of smoking and exogenous processes on non-neoplastic lung tissue.Given a tumor and control sample from the same patient, the tumor sample is used as a readout of mutational signatures, while the control sample is used as a readout of changes in gene expression in non-neoplastic control as a function of mutation signature exposure. The combined analysis of mutational signatures and gene expression with ECoSigClust uncovers functional changes in gene expression (**upper panel**), while the analysis of these signatures leveraging CIBERSORTx uncovers changes in cellular composition (**lower panel**) and sheds light on their correlation with exposures to exogenous processes.

In this paper, we explore the application of the TCGA data for providing a better understanding of the relation between smoking (and other external processes) and molecular-level changes in the lung. Utilizing mutational signatures, derived from cancer lung tissue, and gene expression, derived from the corresponding normal (non-neoplastic control) samples, we hypothesize that such data can inform about the impact of environmental processes on the function of normal lung tissue (Figure 1). We take two complementary computational approaches in our analysis; First, utilizing an approach developed in a previous study [23], we analyzed the relation between patients’ exposures to mutational signatures and gene expression in control samples. Next, recognizing that chronic changes might be related to cellular reprogramming on the tissue level, we utilized methods to decompose bulk samples into cell type proportions to uncover correlations of signature exposures with changes in epithelial and immune cell type proportions. Our study demonstrates the usefulness of such a joint analysis, recapitulating much of the known associations obtained by previous studies (including results obtained using single cell analyses) and providing additional novel insights. It provides a proof of principle of the utility of mutational signatures to be used as sensors of environmental exposures not only in the context of the mutational landscape of cancer, but also as a measurement of important exogenous influences on non-cancer tissues.

## 2. Results

### 2.1. Properties of Mutational Signatures Observed in LUAD Patients

Smoking is a widely recognized risk factor in the emergence of lung diseases. It is also one of the primary mutagens contributing to the emergence of lung adenocarcinoma (LUAD). Previous studies have identified a specific mutational signature (SBS4) that is uniquely associated with smoking [15] and is not observed in non-smokers [24]. This signature is very similar to the mutational signature induced in vitro by exposing cells to a known tobacco smoke carcinogen benzo[a]pyrene, and was shown to correlate with pack years of smoking [15]. This provides strong evidence that SBS4 is a direct consequence of tobacco carcinogens and presents a unique opportunity to study the relation between environmental exposures, represented by mutational signatures from tumor sequencing, and gene expression from control samples. Importantly, even in the context of LUAD—a cancer type that is related to smoking—information on the smoking status is often missing. Quantification of the signature exposure allows to bypass this issue, providing an unbiased estimate of exposure to smoking. We utilized TCGA LUAD mutation data to infer mutational signatures in individual cancer patients, as described in the Section 4.

In addition to the presence of the SBS4 mutational signature in TCGA LUAD data, the genomes of LUAD patients also harbor five additional COSMIC mutational signatures—SBS1, SBS2, SBS5, SBS13, and SBS40 (Section 4). Three LUAD signatures—SBS1, SBS5, and SBS40, are often referred to as “clock-like” signatures, as their strength is positively correlated with patients’ age in many (but not all) cancer types. However, no such correlation is observed in LUAD patients (Figure S1B). Such loss of correlation with age suggests the existence of other factors that accelerate (or otherwise modify) the accumulation of naturally occurring mutations.

Out of the three clock-like signatures, SBS1 is the best understood. It is assumed to arise due to a spontaneous or enzymatic deamination of 5-methylcytosine during replication. Thus, SBS1 is gained during cell division and its accumulation is accelerated in tumor. Consequently, the  exposure of this signature is used to estimate the timing of the tumor initiating event [25]. Consistent with this interpretation, we found that in LUAD, SBS1 is highly associated with primary tumor grade (*p*-value $<4.8\times 10^{-5}$, Figure S1B).

SBS5 is present in nearly all cancer types but its etiology is less understood. As it is frequently correlated with smoking [26], including in LUAD (Figure S1B), it might be the result of exposure to environmental causes occurring with smoking, but also broadly present in other, smoking-independent, contexts. One potential cause might be the accumulation of mutations due to reactive oxygen species (ROS) that play an important role in environment-related mutagenesis, and are prominently associated with smoking [27,28]. SBS5 has also been previously linked to the NER DNA repair pathway [29], but the exact mechanism remains unknown.

The accumulation of SBS40 mutations with age in some cancer types suggests that it might also be related to environmental factors. This is a recently defined signature, characterized by a relatively uniform distribution of mutation types, similarly to SBS5. This renders its contribution uncertain [18]. In the TCGA LUAD dataset, the signature strength of SBS40 is correlated with the signature strength of SBS4 (Figure S1). Thus, we consider these two signatures together in our analysis.

The two remaining signatures, SBS2 and SBS13, are attributed to mutations introduced by the AID/APOBEC family of cytidine deaminases enzymes. The activity of these enzymes is often related to innate immune response [30]. For example, the strength of these signatures has been shown to correlate with the expression of immune-related genes and pathways [23].

The cause of the over-activity of APOBECs in LUAD is yet to be established, but Alexandrov et al. speculated that the cellular machinery underlying SBS2 and SBS13 can be activated by tobacco smoking, perhaps as a smoking-related inflammatory response [15]. Indeed, it has been observed that cigarette smoke incites a potent inflammatory reaction in the airways and alveoli [31], and, in LUAD data, SBS13 exposure is correlated with smoking status (Figure S1B). However, it is also possible that the immune response is related to the fact that the sample was taken from a cancer patient, even if it is from a non-neoplastic lung. In what follows, we will attempt to shed more light on this distinction.

In summary, the mutational signatures observed in LUAD can be divided into three groups: smoking-associated (SBS4, SBS5, SBS40), immune-related (SBS2, SBS13) and the tumor growth-related signature (SBS1).

### 2.2. Pathway-Based Analysis and Relation between Signature Exposures and Gene Expression in Control Samples

**Figure 2:**
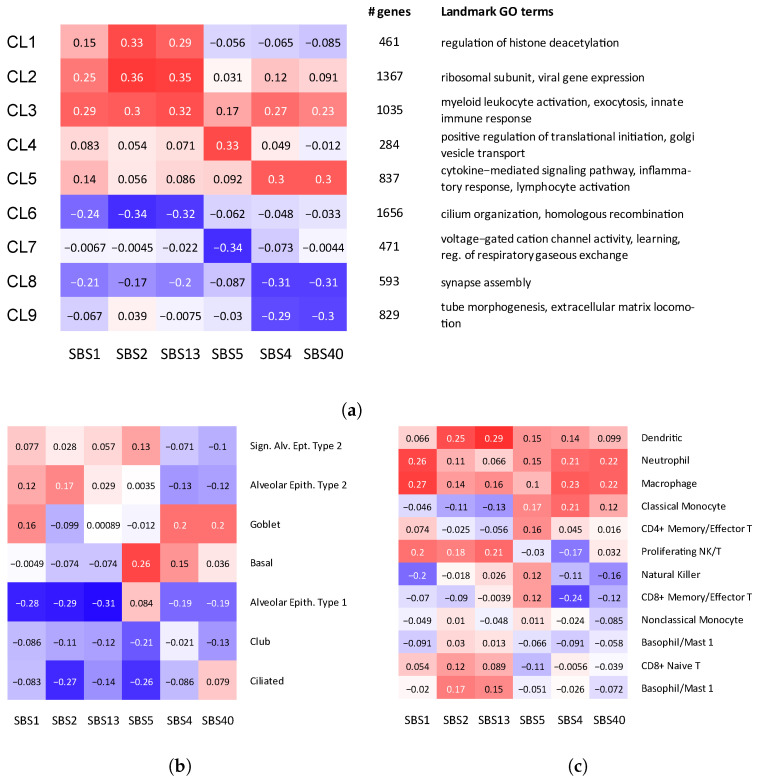
Correlation between mutational signatures and cell type composition and gene expression. (**a**) ECoSigClust clusters, based on the correlation between mutational signatures and gene expression. Genes having a significant correlation with at least one mutational signature (p<0.05) are included in the clustering. The heat map shows the mean correlation between signature and expression among all genes in the cluster (left). For each cluster, the number of genes and representative GO terms enriched in the cluster genes are also shown. (**b**,**c**) Correlation between mutational signatures and cell composition. Bulk expression counts are decomposed into different cell types using CIBERSORTx, and Spearman correlation coefficients are shown for (**b**) epithelial cells and (**c**) immune cells, separately.

First, we asked if mutational signatures can reveal how smoking and other mutagenic processes identified in LUAD interact with gene expression in non-cancer control samples. In an attempt to understand the impact of external mutagens on molecular processes in cells, we utilized the approach developed in a previous study [23] and identified clusters of genes whose expression is correlated with different combinations of signatures (Figure 2a and Table S1). More specifically, we selected genes whose expression is significantly correlated with the strength of at least one mutational signature (p<0.05), and clustered the genes based on their correlation patterns with mutational signatures. We refer to this clustering procedure as ECoSigClust (**e**xpression **co**rrelated **sig**nature **clust**ering).

Gene ontology (GO) enrichment analysis of the clusters obtained by ECoSigClust revealed that the clusters are enriched with specific GO terms, providing insights into the interactions between signatures and molecular pathways. In addition, we analyzed the cluster assignment of known markers of specific lung cell types. Guided by the observations from this analysis, we further explored the association between exposure to exogenous processes and changes in cell-type composition in the lung in the following Section (Section 2.3).

#### 2.2.1. Exposure to Smoking Signature Is Correlated with Increased Inflammatory Response in Non-Cancer Lung Tissue and Elevated Expression of the PD-L1 Immune Checkpoint Gene

The cluster with the strongest positive correlation with the smoking-specific signature SBS4 (and thus with SBS40), which we call *smoking-specific cluster* (CL5, Figure 2a), includes 837 genes, enriched with the cytokine-mediated signaling pathway ($p<10^{-13}$), inflammatory response ($p<10^{-13}$) and cell activation ($p<10^{-14}$, Table S2). This is consistent with previous observations that the exposure of epithelial cells to smoking triggers pro-inflammatory response and increases the release of pro-inflammatory cytokines and chemokines [28,32], many of which are included in the cluster. For example, the  cluster includes several chemokines (CCL2, CCL3, CCL4, CCL7, and CCL11), and pro-inflammatory cytokines (Interleukin 1α (IL1A), and tumor necrosis factor (TNF)) (Table S2). Interestingly, the smoking cluster includes MUC5AC, the canonical marker of mucus-producing secretory goblet cells [33,34], suggesting a relation between smoking and goblet cell population. We investigate the relation further in Section 2.3.

Another notable gene in the cluster is GPR15, a chemoattractant receptor for lymphocytes. The expression of GPR15 was previously found to be up-regulated in smokers [35].

Cluster 5 contains the PD-L1 (CD274) gene. The up-regulation of PD-L1 is believed to allow cancers to evade the host immune system. Thus, immune checkpoint inhibitors of PD-L1 are promising tools for cancer immunotherapy [36,37]. The fact that the association of expression of PD-L1 with smoking is observed in non-cancer lung tissue, and is not related to tumor growth (no correlation with SBS1), is of particular importance. Indeed, a recent experimental study demonstrated that cigarette smoke and the carcinogen benzo(a)pyrene (BaP) induce PD-L1 expression on lung epithelial cells [11].

Finally, Cluster 5 also includes the APOBEC3B gene, which is known to induce mutations related to the emergence of mutational signatures (SBS2 and SBS13). The fact that APOBEC3B belongs to the smoking cluster, rather than a cluster associated with signatures SBS2 and SBS13, suggests that over-activity of this APOBEC enzyme is likely to be triggered by an inflammatory response to smoking [15]. As for negative correlations, we observe that the exposure of these two signatures (SBS2 and SBS13) is negatively correlated with Clusters 8 and 9, both of which are enriched with cell differentiation and morphogenesis. This negative correlation suggests that smoking may shift the overall epithelial function away from a diversity of cell types with specialized functions, toward a consensus increase in mucus secretion, proliferation, and response to stress.

#### 2.2.2. Strength of SBS5, a Signature Correlated with Smoking but Not Unique to This Mutagen, Is Correlated with Changes in Ciliogenesis

The exposure to signature SBS5 is overall correlated with smoking in many cancers, including LUAD. However, as discussed before, this signature is not unique to smokers. The exposure to this signature is negatively correlated with Cluster 7, which is enriched with genes related to voltage gated cation channel activity and neurotransmitter receptor complex. It is known that these channels are targets of a number of naturally occurring toxins and therapeutic agents, as well as environmental toxicants [38], including nicotine [39]. In addition, the cluster also contains known early transcriptional drivers of ciliogenesis, such as MYB and TP73 (Table S1), consistent with the reports that smoking blocks early ciliogenesis [40,41]. The results discussed in Section 2.3 provide further insights into the relation of this signature and changes in the population of ciliated cells in lung.

#### 2.2.3. Relation between the Strengths of APOEBEC-Related Signatures and Gene Expression

The two APOBEC signatures (SBS2 and SBS13) are positively correlated with the expression of genes in Clusters 1–3, and negatively correlated with Cluster 6. We note that correlation of Clusters 2 and 3 with SBS1 suggests a possible relation to tumor growth, so it is not clear to which extent the activity of this cluster is related to smoking and to which extent the changes in the immune system are triggered by tumor growth. Interestingly, Cluster 2 also includes SFTPB and SFTPC, the genes responsible for encoding pulmonary-associated surfactants secreted by the alveolar cells of the lung and maintaining the stability of pulmonary tissue by reducing the surface tension of fluids that coat the lung (Table S1). Interestingly, we found that the expression of the APOBEC3C gene is correlated with the expression of the immune checkpoint gene PD-1 (PDCD1) (*p*-value <0.0051). The APOBEC3C gene is a member of Cluster 2, suggesting that, in contrast to PD-L1, PD-1 might be stimulated by immune response. Out of the three clusters with positive correlation, Cluster 1 correlated with APOBEC signatures most specifically. GO enrichment analysis of this cluster reveals a relation with the regulation of histone deacetylation (Table S2). While a general relation between immune response and histone deacetylation has been well appreciated [42], the association with APOBEC activity remains to be investigated. GO enrichment analysis of Cluster 6, showing negative correlation with APOBEC signatures, found that this cluster is significantly enriched with cilium. Cluster 6 also includes TUBB1, a marker of ciliated cells. This suggests a link between the number of ciliated cells and APOBEC activity.

### 2.3. Mutational Signatures Reveal Relation between Exposure to Exogenous Processes and a Remodeling of Cell-Type Composition in Lung

The signature-dependent expression changes of MUC5AC, a canonical marker of mucus producing secretory goblet cells, as well as other markers discussed in the previous section, suggest a relation between exposures of mutational signatures and changes in the cell-type composition. Indeed, previous studies reported that exposure to smoking leads to the reprogramming of cell-type composition in lungs [3,43]. Thus, we asked whether examining the relation between the exposures of mutational signatures and cell-type composition can identify such trends and potentially provide additional insights.

To investigate the relation between cell-type composition and mutational signatures, we decomposed the bulk expression data using CIBERSORTx [44] and estimated the cell composition in each sample (see Section 4). Considering epithelial and immune cells separately, we then computed the correlation coefficients between the proportions of cell types (within epithelial and immune cell types, respectively) and the strengths of mutational signatures (Figure 2b,c), which revealed several changes in both epithelial and immune cell-type composition correlated with mutational signature activities.

Among epithelial cell types, the proportion of goblet cells is positively correlated with smoking signatures (SBS4, SBS40), while SBS5 has the strongest correlation with Basal cells (Figure 2b). This is consistent with the previous observation that the exposure to cigarette smoke increases the number of mucous-secreting goblet cells and thus can lead to goblet cell hyperplasia, mucus hypersecretion and promote inflammatory responses [45,46].

The correlation pattern of goblet cells is similar to the pattern of smoking cluster in Figure 2a, supporting the hypothesis that the inflammatory responses are generated by epithelial cells with altered cytokine-mediated signaling pathways in response to smoking exposure. Previous studies found that bronchial epithelial cells exposed to cigarette smoke produced a dose-dependent increase in the expression of MUC5AC, IL8 (also called CXCL8) and TNFα genes [47], all of which belong to the smoking cluster.

Interestingly, exposure of SBS4 and SBS5 is correlated with an increased proportion of Basal cells. Basal cells are located below the surface epithelial cell layer and serve as progenitor cells from which ciliated, secretory, and goblet cells differentiate.

Consistent with the results of the previous section, the proportion of ciliated cells has negative correlations with SBS2 and SBS5. The major function of airway ciliated cells is to mediate the propulsion of mucus gel. Thus, a proper balance between goblet and ciliated cells is required for the correct functioning of lungs. Previous studies indicated that this balance might be disturbed by smoking [3,43] and our results confirm this view, but additionally reveal a contribution of APOBEC-related processes captured by SBS2. Interestingly, the reduction in the number of ciliated cells is also associated with SBS2, suggesting a potential relation of the reduction in the ciliated cell number to APOBEC and immune response, which warrants further investigations.

As for immune cells, we observe that innate immune cells, such as dentritic cells, macrophages, and neutrophiles, have overall positive correlation across all mutational signatures (Figure 2c), including the tumor growth-related SBS1. Interestingly the exposure of smoking signature (SBS4) is associated with reduction in CD8+ cells, suggesting an immunosuppressive effect. A similar effect was previously observed in HNSCC cancer [48].

## 3. Conclusions

Exposure of individuals to environmental factors, such as smoking, might lead to molecular changes within cells and the reprogramming of cellular tissue composition. Such changes might be relevant to human health. Yet, the relations between environmental exposures and the above-mentioned changes are not well understood. One of the challenges in studies of the impact of environmental factors on cellular changes is related to the fact that historical exposure to environmental factors is often difficult to quantify. However, many such adverse environmental factors are mutagenic and leave characteristic mutational signatures.

In this paper, we explored whether a joint analysis of mutational signatures and gene expression of non-cancer samples can provide insights into the impact of mutagenic factors on the expression of genes, pathways, and cellular composition in non-neoplastic lung tissue.

Currently, mutational signatures are the most readily accessible for cancer patients by sequencing tumor samples. We reasoned that even if the signatures are inferred from mutations in cancer cells, exogenous environmental factors act on both the cancer and non-cancer cells. Therefore, in this study, we performed a combined analysis of mutational signatures, obtained from cancer genomes, and gene expression from control samples. The fact that a specific mutational signature, SBS1, is known to be correlated with tumor growth, allowed us to identify relations that might be due to tumor growth response in non-neoplastic lung tissue rather than a direct effect of smoking.

Our signature-based analysis uncovered many interesting insights on how smoking can impact the activities of genes, pathways, and tissue composition in lung. The results of our studies are in good agreement with current knowledge, providing confidence in our approach; see Table 1. Furthermore, our results provide additional insights that were not accessible with previous approaches. For example, previous studies demonstrated that smoking can decrease ciliated cells and increase goblet cells in their proportion [3,49]. By analyzing correlations with mutational signature values rather than binary smoking status, our analysis further revealed that the decrease in the ciliated cells proportion is related to the exposure of the SBS5 signature—a signature known to be correlated with smoking but also occurring in contexts not related to smoking.

The interplay between smoking and immune system that we uncovered is also consistent with current knowledge, although the correlation of SBS1 with one of the two immune related clusters suggests that some of the immune response in the control lung tissue could be contributed by an immune response to cancer.

Knowledge of mutational processes acting on a patient’s genome might also help to develop personalized therapies. For example, signature SBS3 indicates homologous recombination deficiency (HRD), and since the patients with HRD are known to benefit from PARP inhibitor therapy [50], the presence of this signature can be used as a marker for PARP inhibitor therapy [51]. Furthermore, APOBEC signatures have been associated with sensitivity to ataxia telangiectasia and Rad3-related kinase (ATR) in some cancer cell lines, suggesting a potential for targeted therapy [52,53,54]. Interestingly, some studies indicated that a smoking history can have an effect on the efficacy of immune checkpoint inhibitors [55]. Our signature-based analysis points to several different mechanisms that, in addition to high tumor mutation burden, can contribute to this effect. First, we found that the strength of smoking signatures is correlated with the expression of the immune checkpoint gene PD-L1, which might promote immune escape. Next, smoking is associated with a reduction in the proportion of CD8+cells, which can kill transformed tumor cells. Finally, the expression of important immune checkpoint gene PD-1 appears to be (indirectly) associated with APOBEC signatures. These examples illustrate an increasing role that mutational signatures play in identifying treatment options.

Overall, we show that looking at the expression changes through the lenses of mutational signatures provides a new and powerful stepping stone for studying the impact of environmental factors on individual’s health, disease susceptibility, and progression. The smoking-associated mutational signature allowed for an unbiased inference of smoking status, key information that is often missing in collected data. In fact, the analysis provided here would have been under-powered if we restricted the study to control samples with reported smoking status only. Finally, cigarette smoke includes a complex mixture of potentially harmful factors, and both direct and indirect as well as mutational signatures based analyses allow for separating at least some of these factors. However, our analysis has also some limitations. Most importantly, the current understating of the mechanisms of many mutational signatures is incomplete, which can limit the interpretability of our association-based results. In addition, as with any association-based approach, additional experiments and knowledge are required to provide mechanistic explanations of the observed dependencies. Finally, while it is easy to obtain mutational signatures from tumor samples, such an approach is indirect, and it would be desirable to measure the mutations directly in the sample of interest. In future, large-scale single-cell sequencing is likely to enable the robust analysis of mutational signatures in non-cancer tissue.

Despite these limitations, our study shows that the utility of mutational signatures can go beyond cancer studies and shed light on the role of environmental mutagens in chronic molecular level changes in the organism. It also provides an example of how a database collected with the purpose of understanding cancer can provide valuable information for studies not directly related to the disease.

## 4. Methods

### 4.1. Mutational Signatures

We downloaded the TCGA LUAD (lung adenocarcinoma) exome mutation spectra and the exome COSMIC reference mutational signatures, provided by Alexandrov et al. [18], from Synapse (accession numbers: syn11801889 and syn11726602, respectively). We utilized the data from 48 patients with known gene expression data for both cancer and control lung tissue. The statistics on this cohort are provided in Table S3. To determine the predominant signatures being active in LUAD samples, we started with the initial sample exposures to mutational signatures from [18] (version 3.1, June 2020, Synapse accession number: syn11804065). The list of active signatures was refined to remove any rare signatures; namely, we keep only signatures that were present in at least 5% of samples and were responsible for at least 1% of mutations. Next, using such a list of active mutational signatures in LUAD (SBS1, SBS2, SBS4, SBS5, SBS13, SBS40, and SBS45), we determined their sample-specific exposures using the quadratic programming (QP) approach available in the R package—–SignatureEstimation [58]. Signature SBS45 was omitted from the analyses presented in this study, as this signature is likely an artifact due to the 8-oxo-guanine introduced during sequencing (see COSMIC Mutational Signatures website: https://cancer.sanger.ac.uk/signatures/ (accessed on 14 September 2022)).

### 4.2. Expression Data

TCGA LUAD RNAseq expression data were obtained from the Genomic Data Commons Data Portal (https://portal.gdc.cancer.gov/ (accessed on 14 September 2022)) on 5 June 2020. HTseq counts were normalized and variance-stabilizing transformed (vst) using DESeq2 [59]. Only donors that had both gene expression and mutational signature exposures were kept, which resulted in 48 normal samples and 466 tumor samples used in this study.

### 4.3. Clustering

To identify expression-based pathways that are associated with signatures, we used ECoSigClust developed for our previous analysis [23]. Specifically, we first computed Spearman correlation coefficients of the expression level and mutation counts for each pair of genes and mutational signatures. We then selected the genes exhibiting significant correlation with at least one of the mutational signatures; the expression of a gene is considered significantly correlated with a signature if nominal p<0.05. This procedure selected 7533 genes. We then clustered the genes based on their correlation patterns using a consensus K-means algorithm; running K-means clustering 100 times with random start, varying *k* from 5 to 50, and subsequently running hierarchical clustering with the consensus matrix from 100 runs of the K-means algorithm. To determine the optimal cluster number, three different clustering validation metrics—Silhouette Index, Calinski–Harabasz Index, and Davies–Bouldin Index—were used, measuring compactness within clusters and separation between clusters slightly differently. The chosen number of clusters k=9 was based on these metrics (Figure S2) and was kept small for the interpretability of each cluster. GO enrichment analysis was performed using the hypergeometric test for each cluster with all genes included in the clustering as the background to assess the differences among the clusters. The list of genes and enrichment analysis results for all clusters are provided in Tables S1 and S2.

### 4.4. Cell Composition Analysis with CIBERSORTx

HTseq raw counts in bulk expression data for the normal samples from TCGA LUAD dataset were used for the analysis. For each gene, the counts in every sample were normalized by the total sum of counts in that sample, multiplied by 1,000,000. The genes without at least one normalized count with a value greater than 1 were discarded. The *Human Lung Cell Atlas* (HLCA) [60] single-cell reference data containing 42 distinct cell types was obtained in the form of counts from synapse (accession number: syn21560511). As per CIBERSORTx guidelines, the same normalization procedure was used on the single-cell reference data and used as input to CIBERSORTx to impute the cell proportions of the 42 given cell types in the bulk TCGA-Lung expression data.

For two subsets of cell types—epithelial and immune cell types, we computed the Spearman correlation of each imputed cell type’s fraction with the exposures of Signatures 1, 2, 4, 5, 13, and 40. The strength of the correlation and the resulting heatmaps are shown in Figure 2.

## Figures and Tables

**Table 1:** Results of the analysis of the relation between mutational signatures and gene expression in the context of previous studies.

Observation From Mutational Signatures	Supporting Literature
Cluster 2:	
ABOBEC signatures are associated with expression of SFTPB and SFTPC	novel observation
APOBEC might indirectly trigger the expression PD-1	[56]
Cluster 5:	
Smoking triggers pro-inflammatory response and cytokines signaling	[28,32]
Smoking increases MUC5AC expression	[34]
Smoking increases PD-L1 expression	[36]
Smoking increases GPR15 expression	[35]
Cluster 6:	
APOBEC is associated with a reduction in cilium organization	novel observation
Cell-type composition:	
ABOBEC signatures are associated with a reduction in CD8+ cells	[48]
Smoking is associated with increase of goblet cells	[45,46,57],
Smoking is associated with decrease of ciliated cells	[3,40,41]
APOBEC is associated with decrease of ciliated cells	novel observation

## Data Availability

ECoSigClust is available at https://github.com/ncbi/ECoSigClust (accessed on 14 September 2022).

## Supplementary Materials

The following supporting information can be downloaded at: https://www.mdpi.com/article/10.3390/biom12101384/s1, Table S1: Gene membership in clusters of genes whose expression is correlated with different combinations of signatures. Table S2: GO enrichment analysis of expression clusters from Table 1. Table S3: Statics on the cohort used in these study. Four stages refer to the extent of patient’s cancer, the mean cigarettes in per day (CPD), mean age in days, gender and number of samples whose corresponding information is available. The mean values were computed over the samples whose corresponding CPD and Days are available. The last column is the number of patients with the given information. The complete information can be obtained from the TCGA data portal. Figure S1: Spearman correlations (above) and corresponding *p*-values (below) represent the pairwise associations in control samples. Figure S2: Evaluation of clustering for varying *k*’s (the number of clusters) using different metrics.
