# Supplementary material for: Mutational Signatures as Sensors of Environmental Exposures: Analysis of Smoking-Induced Lung Tissue Remodeling
Source: Biomolecules. 2022 Sep 27;12(10):1384. doi: 10.3390/biom12101384 (PMC9599238; doi:10.3390/biom12101384)
Supplement: Supplementary file 1 [file biomolecules-12-01384-s001.zip › biomolecules-1878601-supplementary/SupplementaryFigures.pdf]

Supplementary Information
Mutational Signatures as Sensors of Environmental Exposures: Analysis of Smoking-induced Lung Tissue Remodeling

Yoo-Ah Kim*, Ermin Hodzic*, Bayarbaatar Amgalan,
Ariella Saslafsky, Damian Wojtowicz, and Teresa M. Przytycka+

National Center for Biotechnology Information, National Library of Medicine,
National Institutes of Health, Bethesda, MD 20894, USA

Supplemental Figures

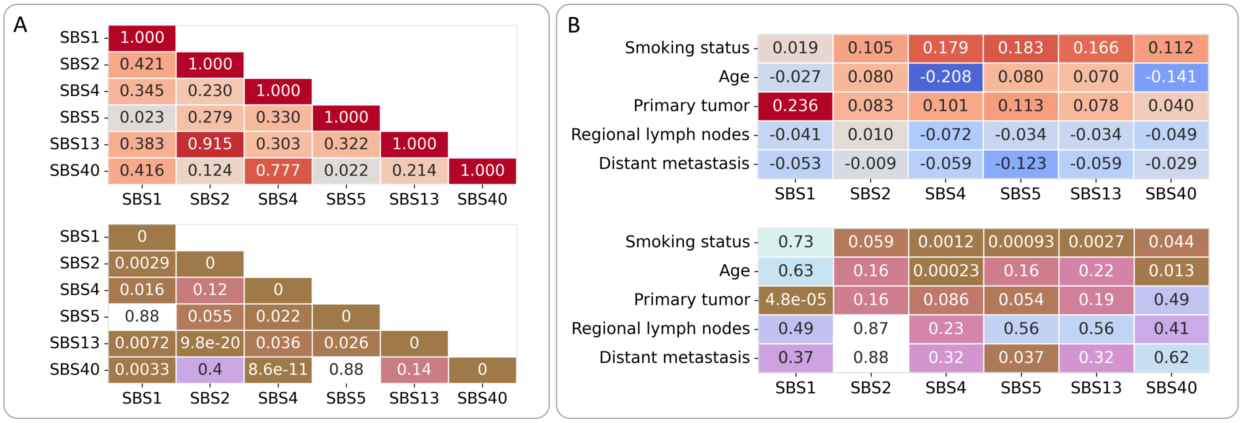

Figure S1: **Spearman correlations (above) and corresponding P-values (below) represent the pairwise associations in control samples.** A. Correlations between signature exposures. B. Correlations between exposure of each signature and smoking status, age and pathological status of patients.

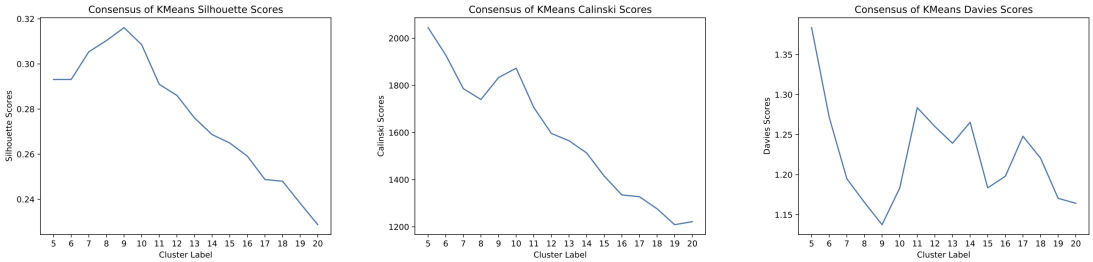

(a) (b) (c)

Figure S2: **Evaluation of clustering for varying k's (the number of clusters) using different metrics:** (a) Silhouette Index, (b) Calinski-Harabasz Index, and (c) Davies-Bouldin Index.
